# Complete Genome Sequence of *Luteitalea* sp. Strain TBR-22

**DOI:** 10.1128/mra.00455-21

**Published:** 2022-02-17

**Authors:** Kyosuke Yamamoto, Yasuko Yoneda, Ayaka Makino, Yasuhiro Tanaka, Xian-Ying Meng, Junko Hashimoto, Kazuo Shin-ya, Noriyuki Satoh, Manabu Fujie, Tadashi Toyama, Kazuhiro Mori, Michihiko Ike, Masaaki Morikawa, Yoichi Kamagata, Hideyuki Tamaki

**Affiliations:** a Bioproduction Research Institute, National Institute of Advanced Industrial Science and Technology, Tsukuba, Ibaraki, Japan; b Bioproduction Research Institute, National Institute of Advanced Industrial Science and Technology, Sapporo, Hokkaido, Japan; c Department of Environmental Sciences, Faculty of Life and Environmental Sciences, University of Yamanashi, Kofu, Yamanashi, Japan; d Japan Biological Informatics Consortium, Tokyo, Japan; e Cellular and Molecular Biotechnology Research Institute, National Institute of Advanced Industrial Science and Technology, Tokyo, Japan; f Okinawa Institute of Science and Technology Graduate University, Okinawa, Japan; g Department of Civil and Environmental Engineering, Faculty of Engineering, University of Yamanashi, Kofu, Yamanashi, Japan; h Division of Sustainable Energy and Environmental Engineering, Graduate School of Engineering, Osaka University, Suita, Japan; i Graduate School of Environmental Science, Hokkaido University, Sapporo, Hokkaido, Japan; j Faculty of Life and Environmental Sciences, University of Tsukuba, Tsukuba, Ibaraki, Japan; k Microbiology Research Center for Sustainability, University of Tsukuba, Tsukuba, Ibaraki, Japan; l Biotechnology Research Center, The University of Tokyo, Tokyo, Japan; Indiana University, Bloomington

## Abstract

We report a complete genome sequence of a novel bacterial isolate, strain TBR-22, belonging to the class *Vicinamibacteria* of the phylum *Acidobacteria*, which was isolated from duckweed fronds. The genome expands our knowledge of the lifestyle of this abundant but rarely characterized phylum.

## ANNOUNCEMENT

Duckweeds are commonly observed freshwater aquatic plants and have been shown to harbor unique microbial communities on their body surface (1, 2). With efforts to isolate novel bacterial lineages from a duckweed-associated microbial community, we successfully isolated a bacterial strain, TBR-22, belonging to the phylum *Acidobacteria* (3). Briefly, isolation was performed with wild duckweeds (*Lemnoideae* spp.) collected from rice paddies located in Ibaraki Prefecture, Japan. Duckweeds were washed and then sonicated with sterile distilled water (SDW), followed by serial dilution with SDW and inoculation on 2.0% (wt/vol) agar plates of diluted tryptic soy broth supplemented with phosphate buffer, vitamin mixture, and basal salt solution (modified diluted tryptic soy broth [mDTS]). The phylum *Acidobacteria* is known to be one of the most abundant bacterial lineages in soils and is also found in various natural environments, including terrestrial freshwater, sediments, and terrestrial plants (4, 5). Despite its wide distribution in natural environments, physiological and ecological characterizations of *Acidobacteria* have not been extensively performed due to its fastidious and difficult-to-culture nature, as only 61 species in this phylum have been validly described to date (4, 5).

Genomic DNA was extracted from cells grown in mDTS at 30°C under static conditions by chemical and enzymatic procedures, as described previously (6), or by using the MagAttract high-molecular-weight (HMW) DNA kit (Qiagen). Library preparation and sequencing were performed by using commercial kits according to the manufacturers’ instructions (Table 1). A total of 1.53 million paired-end reads and 6.32 million mate-pair reads were obtained with the Illumina MiSeq system, and 15 thousand single long reads (mean length, 11,446 bp) were obtained with the Oxford Nanopore Technologies MinION system. Read quality control was performed by FastQC version 0.11.5 (7). Hybrid genome assembly was performed by hybridSPAdes version 3.13.0 (8) in KBase (9) with default settings, and a single scaffold was obtained. Closing of two assembly gaps and genome circularization were performed by Sanger sequencing. The DDBJ Fast Annotation and Submission Tool (DFAST) pipeline version 1.2.13 (10) was used for structural annotation of the TBR-22 genome with following programs: MetaGeneAnnotator version 2008/08/19 (11) for coding sequences, Barrnap version 0.8 (12) for rRNAs, ARAGORN version 1.2.38 (13) for tRNAs, and CRT version 1.2 (14) for CRISPRs.

**TABLE 1:** Library preparation kits and sequencing platforms

Sequencing type	Library prepn kit(s)	Sequencing platform
Illumina short-read sequencing	KAPA HyperPrep kit (for Illumina) (Kapa Biosciences) and Illumina Nextera mate-pair library prepn kit (Illumina)^*a*^	Illumina MiSeq system (paired-end 2 × 300 bp)
MinION long-read sequencing	Rapid sequencing kit (Oxford Nanopore Technologies)	MinION system (R9.4 flow cell)

a Insert lengths for mate-pair libraries were 3 kb and 8 kb.

The complete genome of TBR-22 consists of a 6,468,984-bp-long chromosome, with a G+C content of 70.5%; 5,364 predicted protein-coding DNA sequences, 55 tRNAs, a single set of 5S/165S/23S rRNAs, and no CRISPRs were identified. Genome completeness was estimated with CheckM version 1.1.3 (15), and the genome was determined to be 96.56% complete and 6.64% redundant and to have 4.55% strain heterogeneity. Taxonomic assignment by the Genome Taxonomy Database Toolkit (GTDB-Tk) version 0.1.4 (16) placed TBR-22 within the genus *Luteitalea* in the phylum *Acidobacteriota* (*Acidobacteria*), but it was not assigned a species. The genome obtained can largely contribute to understanding of the ecophysiology of *Acidobacteria*, especially in interactions with aquatic plants, which have been less well investigated than those with terrestrial plants.

### Data availability.

The genome and raw sequences have been deposited in DDBJ/ENA/GenBank under accession number AP024452 and in the DDBJ Sequence Read Archive (DRA) under accession numbers DRA011791 and DRA013041, respectively.

## References

[1] Tanaka Y, Tamaki H, Matsuzawa H, Nigaya M, Mori K, Kamagata Y. 2012. Microbial community analysis in the roots of aquatic plants and isolation of novel microbes including an organism of the candidate phylum OP10. Microbes Environ 27:149–157. doi:10.1264/jsme2.me11288. 22791047 PMC4036017

[2] Tanaka Y, Matsuzawa H, Tamaki H, Tagawa M, Toyama T, Kamagata Y, Mori K. 2017. Isolation of novel bacteria including rarely cultivated phyla, *Acidobacteria* and *Verrucomicrobia*, from the roots of emergent plants by simple culturing method. Microbes Environ 32:288–292. doi:10.1264/jsme2.ME17027. 28740039 PMC5606700

[3] Yoneda Y, Yamamoto K, Makino A, Tanaka Y, Meng X-Y, Hashimoto J, Shin-Ya K, Satoh N, Fujie M, Toyama T, Mori K, Ike M, Morikawa M, Kamagata Y, Tamaki H. 2021. Novel plant-associated *Acidobacteria* promotes growth of common floating aquatic plants, duckweeds. Microoganisms 9:1133. doi:10.3390/microorganisms9061133. PMC8225144 34074043

[4] Kielak AM, Barreto CC, Kowalchuk GA, van Veen JA, Kuramae EE. 2016. The ecology of *Acidobacteria*: moving beyond genes and genomes. Front Microbiol 7:744. doi:10.3389/fmicb.2016.00744. 27303369 PMC4885859

[5] Kalam S, Basu A, Ahmad I, Sayyed RZ, El-Enshasy HA, Dailin DJ, Suriani NL. 2020. Recent understanding of soil acidobacteria and their ecological significance: a critical review. Front Microbiol 11:580024. doi:10.3389/fmicb.2020.580024. 33193209 PMC7661733

[6] Komatsu M, Komatsu K, Koiwai H, Yamada Y, Kozone I, Izumikawa M, Hashimoto J, Takagi M, Omura S, Shin-Ya K, Cane DE, Ikeda H. 2013. Engineered *Streptomyces avermitilis* host for heterologous expression of biosynthetic gene cluster for secondary metabolites. ACS Synth Biol 2:384–396. doi:10.1021/sb3001003. 23654282 PMC3932656

[7] Andrews S. 2010. FastQC: a quality control tool for high throughput sequence data. http://www.bioinformatics.babraham.ac.uk/projects/fastqc.

[8] Antipov D, Korobeynikov A, McLean J, Pevzner P. 2016. hybridSPAdes: an algorithm for hybrid assembly of short and long reads. Bioinformatics 32:1009–1015. doi:10.1093/bioinformatics/btv688. 26589280 PMC4907386

[9] Arkin AP, Cottingham RW, Henry CS, Harris NL, Stevens RL, Maslov S, Dehal P, Ware D, Perez F, Canon S, Sneddon MW, Henderson ML, Riehl WJ, Murphy-Olson D, Chan SY, Kamimura RT, Kumari S, Drake MM, Brettin TS, Glass EM, Chivian D, Gunter D, Weston DJ, Allen BH, Baumohl J, Best AA, Bowen B, Brenner SE, Bun CC, Chandonia J-M, Chia J-M, Colasanti R, Conrad N, Davis JJ, Davison BH, DeJongh M, Devoid S, Dietrich E, Dubchak I, Edirisinghe JN, Fang G, Faria JP, Frybarger PM, Gerlach W, Gerstein M, Greiner A, Gurtowski J, Haun HL, He F, Jain R, Joachimiak MP, Keegan KP, Kondo S, Kumar V, Land ML, Meyer F, Mills M, Novichkov PS, Oh T, Olsen GJ, Olson R, Parrello B, Pasternak S, Pearson E, Poon SS, Price GA, Ramakrishnan S, Ranjan P, Ronald PC, Schatz MC, Seaver SMD, Shukla M, Sutormin RA, Syed MH, Thomason J, Tintle NL, Wang D, Xia F, Yoo H, Yoo S, Yu D. 2018. KBase: the United States Department of Energy Systems Biology Knowledgebase. Nat Biotechnol 36:566–569. doi:10.1038/nbt.4163. 29979655 PMC6870991

[10] Tanizawa Y, Fujisawa T, Nakamura Y. 2018. DFAST: a flexible prokaryotic genome annotation pipeline for faster genome publication. Bioinformatics 34:1037–1039. doi:10.1093/bioinformatics/btx713. 29106469 PMC5860143

[11] Noguchi H, Taniguchi T, Itoh T. 2008. MetaGeneAnnotator: detecting species-specific patterns of ribosomal binding site for precise gene prediction in anonymous prokaryotic and phage genomes. DNA Res 15:387–396. doi:10.1093/dnares/dsn027. 18940874 PMC2608843

[12] Seemann T. 2013. Barrnap 0.7: rapid ribosomal RNA prediction. https://github.com/tseemann/barrnap.

[13] Laslett D, Canback B. 2004. ARAGORN, a program to detect tRNA genes and tmRNA genes in nucleotide sequences. Nucleic Acids Res 32:11–16. doi:10.1093/nar/gkh152. 14704338 PMC373265

[14] Bland C, Ramsey TL, Sabree F, Lowe M, Brown K, Kyrpides NC, Hugenholtz P. 2007. CRISPR Recognition Tool (CRT): a tool for automatic detection of clustered regularly interspaced palindromic repeats. BMC Bioinformatics 8:209. doi:10.1186/1471-2105-8-209. 17577412 PMC1924867

[15] Parks DH, Imelfort M, Skennerton CT, Hugenholtz P, Tyson GW. 2015. CheckM: assessing the quality of microbial genomes recovered from isolates, single cells, and metagenomes. Genome Res 25:1043–1055. doi:10.1101/gr.186072.114. 25977477 PMC4484387

[16] Chaumeil P-A, Mussig AJ, Hugenholtz P, Parks DH. 2019. GTDB-Tk: a toolkit to classify genomes with the Genome Taxonomy Database. Bioinformatics 36:1925–1927. doi:10.1093/bioinformatics/btz848. PMC7703759 31730192

